# The combinations of cefazolin with linezolid and cefazolin with clindamycin are indifferent against methicillin-susceptible *Staphylococcus aureus*

**DOI:** 10.1128/spectrum.01763-25

**Published:** 2025-09-30

**Authors:** Madeline Mellett, Julia Van Riel, Rachel Corsini, Sarah Satola, Alexander Lawandi, Chelsea Caya, Sebastiaan J van Hal, Todd C. Lee, Jesse Papenburg, Cedric P. Yansouni, Ahmed Babiker, Matthew P. Cheng

**Affiliations:** 1 Department of Microbiology and Immunology, McGill University 5620, Montreal, Quebec, Canada; 2 Division of Infectious Diseases, Department of Medicine, Emory School of Medicine 12244, Atlanta, Georgia, USA; 3 Research Institute of the McGill University Health Centre 54473, Montreal, Quebec, Canada; 4 Department of Critical Care Medicine, McGill University Health Centre 54473, Montreal, Quebec, Canada; 5 Divisions of Infectious Diseases and Medical Microbiology, McGill University Health Centre 54473, Montreal, Quebec, Canada; 6 Department of Infectious Diseases and Microbiology, NSW Health Pathology, Royal Prince Alfred Hospital 2205, Sydney, New South Wales, Australia; 7 Central Clinical School, University of Sydney 4334, Sydney, New South Wales, Australia; 8 Division of Pediatric Infectious Diseases, Department of Pediatrics, McGill University Health Centre 54473, Montreal, Quebec, Canada; 9 J.D. MacLean Centre for Tropical and Geographic Medicine, McGill University 5620, Montreal, Quebec, Canada; Seton Hall University, South Orange, New Jersey, USA

**Keywords:** *Staphylococcus aureus*, synergy, MSSA

## Abstract

Antimicrobial combinations resulting in synergy against *Staphylococcus aureus* are desirable for improving clinical efficacy in the treatment of severe infections caused by this pathogen. Despite an existing biological rationale for the combination of cefazolin and linezolid for methicillin-susceptible *S. aureus* (MSSA), this combination has yet to be evaluated for *in vitro* synergy. We employed the checkerboard and E-test methods of synergy assessment to evaluate cefazolin in combination with linezolid against 19 clinical MSSA isolates from two centers and compared the findings to cefazolin in combination with another protein synthesis inhibitor, clindamycin. Synergy was determined using the fractional inhibitory concentration index (FICI) through both the checkerboard and combined E-test assays. Combinations of cefazolin with linezolid and clindamycin demonstrated indifference through both methods (median checkerboard FICI of 1.11 and 1.25 and median E-test FICI of 0.99 and 0.88, respectively). Although the studied combinations were not synergistic, results of both assays were categorically consistent and suggest that the E-test is a robust method for evaluating synergy in *S. aureus* isolates.

**IMPORTANCE:**

The combinations of cefazolin and linezolid and cefazolin with clindamycin are potentially clinically relevant combinations for the treatment of severe *S. aureus* infections. However, the synergistic effects of these combinations against clinical MSSA isolates have not been previously determined. We performed checkerboard and E-test synergy testing and demonstrated that these combinations were indifferent for MSSA isolates. Although synergy was not identified for these combinations, the methods of synergy assessment agreed, highlighting the potential benefit for the combined gradient diffusion assay in future synergy assessment. Due to ease of use and categorical agreement with the checkerboard method in this study, this method may be beneficial for further implementation in synergy testing, leading to the identification of synergistic combinations that may improve treatment success for severe *S. aureus* infections.

## INTRODUCTION

*Staphylococcus aureus* is an important human pathogen, responsible for a variety of infections including skin and soft tissue infections, as well as severe life-threatening diseases such as bacteremia, endocarditis, pneumonia, and sepsis (1). Persistent high mortality rates for severe infections warrant continued investigation into novel treatment strategies that can improve patient outcomes (2). One strategy to address this need is combining existing antibiotics to achieve synergy (3–7).

Despite extensive studies investigating combinations of antibiotics for *S. aureus*, few existing studies have evaluated the combination of linezolid with β-lactams against methicillin-susceptible *S. aureus* (MSSA) (8–13), with combinations tested including linezolid with meropenem, imipenem, ampicillin-sulbactam, oxacillin, ceftaroline, and ceftobiprole. Linezolid has potential for use in combination therapy for *S. aureus* because it is well tolerated, penetrates tissues effectively, and has a favorable safety profile, especially when used for short-course therapy (14, 15). Furthermore, linezolid is active against both methicillin-resistant *S. aureus* (MRSA) and MSSA, with limited resistance currently identified (16–18). Leveraging the findings of our recent systematic review on synergy for *S. aureus*, the combination of cefazolin with linezolid has not yet been evaluated *in vitro* for MSSA (19).

The combination of cefazolin and linezolid has potential important clinical value for the treatment of MSSA infections as cefazolin is a first-generation cephalosporin frequently employed to treat MSSA (20, 21). From an *in vitro* standpoint, linezolid has a distinct mechanism of action than β-lactams. Linezolid is a protein synthesis inhibitor within the oxazolidinone class of antibiotics that exerts its effect through binding to the 50S ribosomal subunit (22). Linezolid can also address certain pitfalls of β-lactam use. The activity of linezolid is not affected by inoculum size, and unlike β-lactams, which become less effective against nonreplicating bacteria, may have comparatively greater activity against dormant bacteria (23, 24).

 In this study, we evaluated the synergistic potential of the combination of linezolid and cefazolin against 19 clinical MSSA isolates through both the checkerboard and the combined gradient diffusion (E-test) method of synergy assessment. The combination of cefazolin with clindamycin, a protein-synthesis inhibitor with similar activity as linezolid, was used as a comparator and was assessed for synergy using the same methods (25).

## MATERIALS AND METHODS

### Bacterial strains

Nineteen unique MSSA isolates were used in this study. Nine unique isolates were obtained from the *Staphylococcus aureus* Network Adaptive Platform (SNAP) (Strains 1–9), and were collected from Australia between 2014 and 2015. Three replicate isolates were available for each isolate type and were used in replicate MIC and synergy testing. Isolates are not linked to participants enrolled in the SNAP trial. Ten clinical blood stream isolates were obtained from Emory’s Investigational Clinical Microbiology Core (ICMC) laboratory (Strains 0013, 0073, 0048, 0137, 0022, 0180, 0021, 0314, 0670, and 0861), between 2022 and 2023. SNAP isolates were confirmed as *S. aureus* by Maldi-TOF, and penicillin susceptibility was determined using *blaZ* PCR. Six isolates were confirmed as penicillin-resistant (Strains 1, 3, 6, 7, S, and 9). Emory isolates were confirmed as *S. aureus* by Maldi-TOF. Isolates were stored in 12% glycerol with Cation-Adjusted Mueller-Hinton Broth (CAMHB) and frozen at −80°C prior to use. ATCC 25923 was used throughout all experiments as a control.

### MIC determination

The minimum inhibitory concentration (MIC) of the isolates and controls was determined using the gold standard broth microdilution (BMD) method (26). Antibiotics were serially diluted into 96-well plates containing 50 µL CAMHB to achieve a concentration range of 16 μg/mL–0.0156 μg/mL. Then, 50 µL of 1 × 10^6^ CFU/mL bacteria was added following overnight incubation on Columbia Blood Agar plates (SNAP isolates) or TSA blood agar (Emory isolates) at 37°C for 20 hours. SNAP isolates were incubated for 18–20 hours at 37°C, incubated with shaking at 200 RPM for 15 minutes at 37°C, and then returned to the static 37°C incubator for an additional 2 hours. Growth was measured using the Tecan Infinite M200 Pro at OD_600_ and validated visually. Emory isolates were incubated for 18–20 hours at 37°C, and then MIC was determined visually. The MIC of SNAP isolates was interpreted using the E-test, as recommended by the manufacturer (LioFilChem Inc.). Isolates were incubated overnight at 37°C in CAMHB and diluted to 0.5 McFarland (McF) using the McF densitometer DEN-1 (Grant-Bio). Sterile cotton swabs were used to plate dense bacterial lawns on Mueller-Hinton Agar (MHA) plates. E-test strips were then placed onto the plates and incubated at 37°C for 20 hours. Zones of inhibition were evaluated, and MIC was recorded. E-test MICs were not determined for Emory isolates.

### Synergy testing

**Fig 1:**
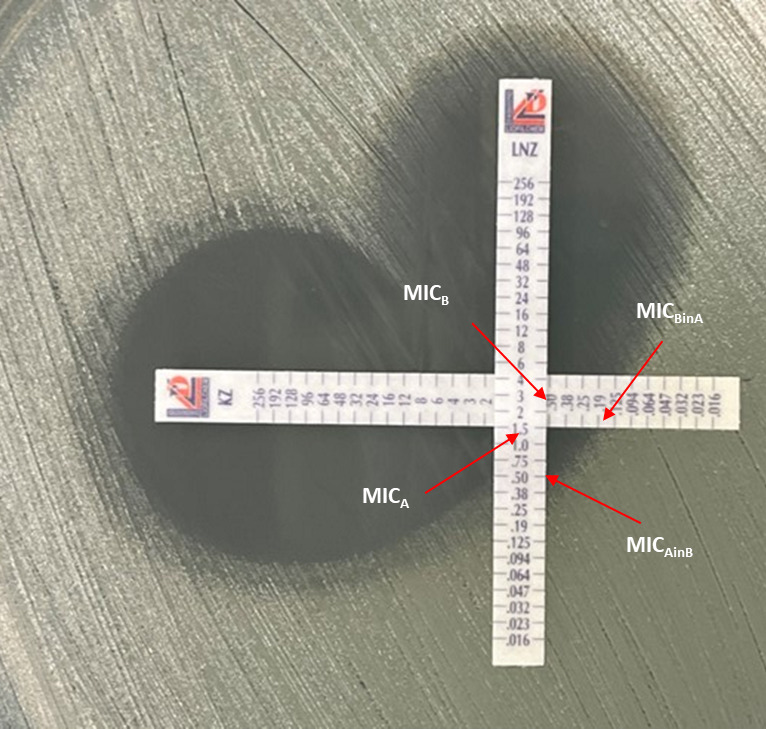
Comined antimicrobial gradient strip test for Synergy. Strips were placed at a 90° angle such that the MICs of each antibiotic are intersecting. Arrows are pointing to the MIC of each antibiotic (MICA = 1.5 µg/mL; MICB = 0.50 µg/mL) and the MIC of each antibiotic in the presence of the other (MICAinB = 0.50 µg/mL; MICBinA = 0.19 µg/mL).

Checkerboard assays were prepared for each isolate using the microtiter broth dilution technique. Antibiotics were serially diluted to achieve the MIC of the corresponding isolate to be tested in the middle of the plate for each antibiotic. Isolates were cultured overnight on Columbia blood agar (SNAP isolates) or TSA blood agar (Emory isolates), and individual colonies were diluted in CAMHB. SNAP isolates were first diluted to 0.5 McF and then further diluted to achieve ~7.5×10^5^ CFU/mL. Emory isolates were diluted to a 0.5 McF and then diluted to achieve ~7.5×10^5^ CFU/mL. The CFU/mL for all checkerboards were confirmed and ranged from 1.0 × 10^5^ to 5.7 × 10^6^ CFU/mL. Then, 100 µL of bacteria was added to all wells of the checkerboard containing 100 uL of antibiotics excluding one well lacking antibiotics, serving as a contamination control. For SNAP isolates, checkerboard plates were incubated for 20 hours at 37°C, followed by shaking for 15 minutes at 200 RPM and 37°C. The plates were returned to the 37°C incubator for 2 hours. Bacterial growth was measured using the Tecan infinite M200 Pro at OD_600_. Emory isolates were incubated with shaking for 15 minutes and then returned to the static 37°C incubator for 20 hours. OD was measured using the BioTek Eon, Gen5 software. The fractional inhibitory concentration index (FICI) was then calculated according to the NCLSS criteria using the following formula: ΣFIC = FIC_A_ + FI_CB_ where FIC_A_ = MIC_(A in the presence of B)_ / MIC_(A alone)_ , FIC_B_ = MIC_(B in the presence of A)_ / MIC_(B alone)_ (27). An FIC index ≤0.5 is consistent with synergy, 0.5 < FIC ≤ 4 with indifference, and an FIC >4 with antagonism (27). For each checkerboard plate, the mean, minimum, and maximum FICI were determined for all replicates. Mean FICI for each plate was used to determine synergy and is hereafter referred to as FICI. Mean, minimum, and maximum FICI results are presented as the mean of all replicates tested. In parallel, the combined E-test assay was performed for synergy determination (28). Isolates were diluted from individual colonies from overnight cultures to a 0.5 McF standard using the McF densitometer DEN-1 (Grant-Bio) for SNAP isolates or the Vitek DensiCheck for Emory isolates. A sterile cotton swab was used to create a lawn of bacteria on Mueller Hinton or CAMBH agars. E-test strips were placed in a 90° cross formation intersecting at the previously determined MICs (Fig. 1). E-test plates were incubated at 37°C for 20 hours. For both groups of isolates, MICs in combination were evaluated visually, FIC was calculated as described previously, and the synergy result was determined.

A minimum of three independent synergy tests were performed for each clinical isolate type. Results are presented as mean or median of the synergy assessments for each unique isolate.

## RESULTS

### MICs

The MICs for 19 clinical MSSA isolates and the control isolate ATCC 25923 were determined through both the BMD and E-test method. According to the BMD method, the MICs of ATCC 25923 were 0.25 µg/mL for cefazolin, 0.0625 µg/mL for clindamycin, and 2 for linezolid, respectively. Through the E-test method, the MICs were 0.5 µg/mL for cefazolin, 0.125 µg/mL for clindamycin, and 3 µg/mL for linezolid. Clinical isolates were susceptible to all antibiotics. The median cefazolin MIC by BMD method of all isolates tested was 0.25 µg/mL (range 0.125–1 μg/mL), the median MIC of clindamycin was 0.125 µg/mL (range 0.0625–0.25μg/mL), and the median MIC of linezolid was 2 µg/mL (range 1–4 μg/mL) (Table 1).

**TABLE 1:** Minimum inhibitory concentrations of 19 clinical MSSA isolates according to the broth microdilution method^*a*^

	Minimum Inhibitory concentration (μg/mL)				
	≤ 0.094 – ≥0.00625	≤ 0.25 – >0.094	≤1–0.25	≤2–1	4 – >2
CEF BMD	0/19 (0%)	6/19 (31.6%)	13/19 (68.4%)	0/19 (0%)	0/19 (0%)
CLI BMD	2/19 (10.5%)	17/19 (89.5%)	0/19 (0%)	0/19 (0%)	0/19 (0%)
LIN BMD	0/19 (0%)	0/19 (0%)	0/19 (0%)	12/19 (63.2%)	7/19 (36.8%)

^*a*^ CEF: cefazolin, CLI: clindamycin, LIN: linezolid, BMD: Broth Microdilution.

### Checkerboard assay for synergy

Determination of synergy through the checkerboard method was similar for both combinations of cefazolin and linezolid and cefazolin and clindamycin. Cefazolin and linezolid were indifferent for all isolates (0.5 < FICI < 4), and mean FICI for each isolate is presented in Table 2. The median FICI across isolates for this combination was 1.11 (ranging from 0.87 to 1.33). The combination of cefazolin and clindamycin was also indifferent with a median FICI of 1.25 (1.03–1.69). The minimum and maximum FICI for each isolate can be seen in Table S1. Synergy nor antagonism was demonstrated for any isolate for either combination.

**TABLE 2:** Results of synergy assessment through both the checkerboard and E-test approaches

	Cefazolin and linezolid				Cefazolin and clindamycin			
	Mean FICI^*a*^ checkerboard	Range, standard deviation (SD)	Mean FICI E-test	Range, SD	Mean FICI checkerboard	Range, SD	Mean FICI E-test	Range, SD
ATCC 25923	1.18	1.12–1.23, 0.08	1.42	NA^*b*^	1.46	1.42–1.48, 0.04	1.25	NA
Strain 1	1.10	0.75–1.44, 0.27	1.02	0.71–2.00, 0.49	1.12	0.91–1.43, 0.20	1.31	0.64–2.00, 0.61
Strain 2	1.28	0.99–1.36, 0.14	1.24	1.00–2.00, 0.39	1.37	1.26–1.43, 0.07	0.94	0.76–1.16, 0.16
Strain 3	1.10	0.98-1.25, 0.10	0.96	0.83–1.25, 0.16	1.25	1.04–1.53, 0.16	0.82	0.70–1.25, 0.22
Strain 4	1.19	0.98–1.84, 0.32	0.99	0.83–1.26, 0.16	1.14	0.96–1.39, 0.17	0.98	0.71–1.17, 0.15
Strain 5	0.98	0.69–1.21, 0.20	1.02	0.88–1.17, 0.13	1.41	1.04–1.65, 0.26	1.01	0.88–1.25, 0.14
Strain 6	1.27	1.03–1.62, 0.22	1.08	0.83–1.41, 0.26	1.69	1.23–2.09, 0.31	0.93	0.64–1.18, 0.22
Strain 7	1.33	0.98–1.97, 0.40	0.79	0.67–1.00, 0.13	1.22	1.18–1.23, 0.04	0.86	0.59–1.02, 0.18
Strain 8	1.10	0.98–1.25, 0.10	1.19	0.83–1.32, 0.21	1.15	1.01–1.41, 0.15	1.19	1.00–1.41, 0.16
Strain 9	1.05	0.98–1.22, 0.09	1.28	0.88–2.00, 0.42	1.03	0.90–1.25, 0.13	0.89	0.76–1.01, 0.10
Strain 0013	1.21	1.12–1.26, 0.08	0.92	0.76–1.00, 0.14	1.34	1.12–1.45, 0.19	0.92	0.63–1.51, 0.51
Strain 0073	1.23	1.12–1.31, 0.10	0.88	0.76–1.00, 0.12	1.31	1.12–1.45, 0.17	0.71	0.63–0.88, 0.14
Strain 0048	1.19	1.12–1.23, 0.06	0.92	0.88–1.00, 0.07	1.51	1.35–1.63, 0.14	0.75	0.63–0.88, 0.12
Strain 0137	1.03	0.98–1.12, 0.08	0.84	0.76–0.88, 0.07	1.24	1.12–1.31, 0.10	0.75	0.63–0.88, 0.13
Strain 022	1.11	0.84–1.36, 0.26	1.29	0.88–1.75, 0.44	1.25	1.12–1.39, 0.13	0.92	0.63–1.25, 0.31
Strain 0180	1.13	1.05–1.20, 0.07	0.92	0.88–1.00, 0.07	1.24	1.03–1.36, 0.18	0.76	0.64–0.88, 0.12
Strain 0021	0.87	0.77–1.05, 0.15	1.17	1.00–1.25, 0.14	1.13	1.03–1.25, 0.11	0.88	0.76–1.00, 0.12
Strain 0314	1.11	0.93–1.29, 0.18	1.09	1.00–1.26, 0.15	1.48	1.40–1.59, 0.10	0.84	0.76–1.00, 0.14
Strain 0670	0.96	0.70–1.14, 0.23	0.75	0.63–0.88, 0.13	1.39	1.28–1.45, 0.09	0.59	0.51–0.64, 0.07
Strain 0861	1.06	0.95–1.25, 0.16	0.88	0.88–0.88, 0.00	1.06	0.96–1.25, 0.16	0.75	0.63–0.86, 0.12

^*a*^ Mean FICI represents the average of all determined mean FICIs from each replicate for the corresponding isolates.

^*b*^ NA, not available. E-tests were not performed in replicate for the control strain.

### E-test for synergy

Synergy assessment through the E-test method demonstrated similar findings as the checkerboard method. Indifference was identified for both tested combinations against all clinical isolates. The median FICI according to the E-test method for the combination of cefazolin and linezolid was 0.99 (0.75–1.29). The median FICI for the combination of cefazolin and clindamycin was 0.88 (0.59–1.31). See Table 2 for details of individual isolates. These findings demonstrate that these antimicrobial combinations are not synergistic. Identification of indifference was consistent between E-test and checkerboard, indicating categorical agreement for these methods against these antibiotic combinations although numerical correlation was distinct (r = −0.32, 95% CI:−0.58 to 0.00).

## DISCUSSION

This is the first study assessing the synergistic effect of the combination of cefazolin and linezolid against MSSA. We demonstrated that this combination was indifferent against all isolates through both the checkerboard and E-test methods. The comparator combination of cefazolin and clindamycin demonstrated similar results with both the checkerboard and E-test consistently resulting in indifference.

 Identification of indifference for these combinations may be attributable to the mechanism of action of the antibiotics when used in combination. Cephalosporins inhibit cell wall synthesis through interaction with penicillin-binding proteins (29, 30). However, protein synthesis inhibitors including linezolid and clindamycin inhibit protein synthesis (31), which may affect the ability of cephalosporins to bind penicillin-binding proteins. However, this remains to be formally demonstrated for the combinations tested here and was not reflected by identification of antagonism in this study.

These results are consistent with previous synergy assessment of linezolid with other cephalosporins. Romero et al. demonstrated that the combination of linezolid and ceftaroline was poorly synergistic for both methicillin-resistant *S. aureus* (MRSA) and MSSA, with only 1 out of 31 tested isolates demonstrating synergy (FICI ≤0.5) using E-tests in the MIC:MIC ratio (12). The remaining isolates demonstrated additivity/indifference ( >0.5 to ≤4) for this combination (12). A similar study performed by García et al. assessing linezolid and ceftaroline against both MRSA and methicillin and linezolid-resistant *S. aureus* (MLRSA) using E-tests similarly identified synergy in only 1 instance out of 71 tested isolates (32). However, when the combination of ceftriaxone and linezolid was assessed using the checkerboard approach in a separate study of linezolid-susceptible MRSA isolates, synergy (FIC ≤0.5, Median FIC 0.3) was demonstrated against the majority of tested isolates (23/30) (33). Disagreement between these studies may be dependent upon the β-lactam antibiotics or the isolates being assessed rather than the different methods as we demonstrated that the checkerboard and E-test methods were comparable through parallel checkerboard and E-test assessment. Alternatively, synergy is an uncommon phenotype and limited to a subset of the overall *S. aureus* population.

E-test assays for synergy have been limited in their application for synergy testing; however, they offer significant advantages for simplicity and ease of interpretation when compared to the checkerboard and other methods of *in vitro* synergy testing including the time-kill assay (34). Consistent with our findings, White et al. demonstrated agreement between the two methods for several combinations when tested against ATCC 29213. (28) Our study supports this finding by assessing a greater number of isolates and suggests that the E-test should be considered in future synergy assessment to enable evaluation of a greater number of isolates and antibiotic combinations for synergy testing with subsequent translation into a clinical laboratory setting (35).

Our study is limited, in that we only performed our checkerboard assay at one inoculum that is not representative of a high bacterial load. Synergy may instead be evident when assessed using a high bacterial inoculum, at which point β-lactams are less effective as compared to protein synthesis inhibitors (36, 37). Synergy assessment of this combination does not assess the clinical benefit of proposed exotoxin inhibition of protein synthesis inhibitors (38, 39). This effect may be relevant in toxin-mediated infections caused by *S. aureus* (1, 36). Future investigations should employ a high-inocula assay to compare the synergy outcome to a standard inoculum in addition to measuring protein expression for the tested combinations of cephalosporins with linezolid and clindamycin. Another limitation of this study is that the methodology for synergy assessment varied between institutions where synergy testing was conducted. Nevertheless, categorical agreement of indifference at both institutions is compelling that results are comparable, especially considering that different methodologies were employed at both sites.

Taken together, our study highlights that combinations of cefazolin with linezolid or cefazolin with clindamycin are indifferent. However, we demonstrated agreement between checkerboard and E-test methods of synergy determination for many MSSA isolates. Due to the ease of use of the E-test method, it may be pertinent to use this approach for future synergy assessments to more broadly and rapidly evaluate combinations for antimicrobial synergy.

## SUPPLEMENTAL MATERIAL

Supplemental table spectrum.01763-25-s0001.docx FICI min and FICI max for performed checkerboard assays.

ASM does not own the copyrights to Supplemental Material that may be linked to, or accessed through, an article. The authors have granted ASM a non-exclusive, world-wide license to publish the Supplemental Material files. Please contact the corresponding author directly for reuse.
