# Supplementary material for: The combinations of cefazolin with linezolid and cefazolin with clindamycin are indifferent against methicillin-susceptible Staphylococcus aureus
Source: Microbiol Spectr. 2025 Sep 30;13(11):e01763-25. doi: 10.1128/spectrum.01763-25 (PMC12584723; doi:10.1128/spectrum.01763-25)
Supplement: Supplemental table — FICI min and FICI max for performed checkerboard assays. [file spectrum.01763-25-s0001.docx]

**Supplementary Materials**

|  | **Cefazolin and linezolid** | | | | **Cefazolin and clindamycin** | | | |
| --- | --- | --- | --- | --- | --- | --- | --- | --- |
|  | FICI_min_ | FICI_min_ range, SD | FICI_max_ | FICI_max_ range, SD | FICI_min_ | FICI_min_ range, SD | FICI_max_ | FICI_max_ range, SD |
| **ATCC 25923** | 1.02 | 1.00-1.03, 0.02 | 1.34 | 1.25-1.50, 0.18 | 1.02 | 1.00-1.03, 0.02 | 2.19 | 2.13-2.25, 0.09 |
| **Strain 1** | 0.72 | 0.38-1.02, 0.26 | 1.51 | 1.06-2.25, 0.48 | 0.78 | 0.62-1.03, 0.20 | 1.65 | 1.13-2.50, 0.60 |
| **Strain 2** | 0.78 | 0.63-1.00, 0.16 | 2.19 | 2.13-2.25, 0.07 | 1.02 | 1.00-1.03, 0.02 | 2.28 | 2.12-2.50, 0.16 |
| **Strain 3** | 0.88 | 0.75-1.02, 0.14 | 1.44 | 1.13-2.25, 0.42 | 0.96 | 0.75-1.02, 0.10 | 1.88 | 1.25-2.50, 0.52 |
| **Strain 4** | 0.80 | 0.63-1.03, 0.18 | 1.60 | 1.25-2.50, 0.56 | 0.89 | 0.75-1.03, 0.15 | 1.45 | 1.13-2.06, 0.34 |
| **Strain 5** | 0.73 | 0.53-1.00, 0.22 | 1.51 | 1.02-2.25, 0.58 | 1.01 | 0.75-1.50, 0.25 | 1.86 | 1.25-2.50, 0.61 |
| **Strain 6** | 0.86 | 0.56-1.06, 0.21 | 2.10 | 1.50-2.50, 0.33 | 1.10 | 0.75-1.50, 0.25 | 2.46 | 2.25-2.50, 0.10 |
| **Strain 7** | 0.80 | 0.63-1.03, 0.21 | 1.83 | 1.25-3.00, 1.01 | 1.03 | 1.01-1.06, 0.03 | 1.50 | 1.50-1.50, 0.00 |
| **Strain 8** | 0.82 | 0.63-1.03, 0.16 | 1.44 | 1.25-2.13, 0.35 | 0.93 | 0.75-1.03, 0.14 | 1.46 | 1.25-2.25, 0.40 |
| **Strain 9** | 0.70 | 0.56-0.75, 0.08 | 1.44 | 1.25-2.13, 0.35 | 0.76 | 0.53-1.03, 0.21 | 1.27 | 1.12-1.50, 0.12 |
| **Strain 0013** | 1.04 | 1.00-1.06, 0.04 | 1.42 | 1.25-1.50, 0.14 | 1.04 | 1.00-1.06, 0.04 | 1.92 | 1.25-2.25, 0.58 |
| **Strain 0073** | 0.94 | 0.75-1.06, 0.17 | 1.60 | 1.25-2.06, 0.42 | 1.02 | 1.00-1.06, 0.04 | 1.92 | 1.25-2.25, 0.58 |
| **Strain 0048** | 1.02 | 1.00-1.03, 0.02 | 1.42 | 1.25-1.50, 0.14 | 1.04 | 1.03-1.06, 0.02 | 2.42 | 2.25-2.50, 0.14 |
| **Strain 0137** | 0.63 | 0.63-0.63, 0.00 | 1.51 | 1.25-2.03, 0.45 | 1.06 | 1.00-1.06, 1.13 | 1.42 | 1.25-1.50, 0.14 |
| **Strain 022** | 0.86 | 0.56-1.03, 0.26 | 1.50 | 1.13-2.13, 0.54 | 1.02 | 1.00-1.03, 0.02 | 1.67 | 1.25-2.25, 0.52 |
| **Strain 0180** | 0.83 | 0.75-1.00, 0.14 | 1.52 | 1.25-2.06, 0.47 | 0.92 | 0.75-1.00, 0.14 | 1.92 | 1.25-2.25, 0.58 |
| **Strain 0021** | 0.61 | 0.53-0.75, 0.12 | 1.15 | 1.06-1.25, 0.10 | 0.93 | 0.75-1.03, 0.15 | 1.33 | 1.25-1.50, 0.14 |
| **Strain 0314** | 0.67 | 0.63-0.75, 0.07 | 1.76 | 1.13-2.13, 0.55 | 1.05 | 1.03-1.06, 0.02 | 2.33 | 2.25-2.50, 0.14 |
| **Strain 0670** | 0.76 | 0.53-1.00, 0.23 | 1.19 | 1.06-1.25, 0.11 | 1.06 | 1.06-1.06, 0.00 | 2.00 | 1.50-2.25, 0.43 |
| **Strain 0861** | 0.80 | 0.63-1.03, 0.21 | 1.25 | 1.13-1.50, 0.22 | 0.80 | 0.63-1.03, 0.21 | 1.29 | 1.13-1.50, 0.19 |

**Supplementary Table .** Average of Minimum and Maximum FICI for the combinations of both cefazolin and linezolid and cefazolin and clindamycin for each isolate. FICIs are a mean of all replicates for each isolate.
